# Postnatal probiotic supplementation can prevent and optimize treatment of childhood asthma and atopic disorders: A systematic review of randomized controlled trials

**DOI:** 10.3389/fped.2022.956141

**Published:** 2022-08-19

**Authors:** Samuel N. Uwaezuoke, Adaeze C. Ayuk, Joy N. Eze, Chioma L. Odimegwu, Chibuzo O. Ndiokwelu, Ikenna C. Eze

**Affiliations:** ^1^Department of Paediatrics, University of Nigeria Teaching Hospital, Ituku-Ozalla, Enugu, Nigeria; ^2^Department of Pediatrics, College of Medicine, University of Nigeria, Ituku-Ozalla Enugu Campus, Enugu, Nigeria; ^3^Department of Epidemiology and Public Health, Swiss Tropical and Public Health Institute, Allschwil, Switzerland; ^4^University of Basel, Basel, Switzerland

**Keywords:** atopic disease, childhood asthma, gastrointestinal microbiota, prevention, probiotics, therapeutics

## Abstract

**Background:**

Although several randomized controlled trials (RCTs) published over the past 5 years show that prenatal or postnatal probiotics may prevent or optimize the treatment of childhood asthma and atopic disorders, findings from the systematic reviews and meta-analyses of these studies appear inconsistent. More recent RCTs have focused on postnatal probiotics, and linked specific probiotic strains to better disease outcomes.

**Objective:**

This systematic review aimed to determine if postnatal probiotics are as effective as prenatal probiotics in preventing or treating childhood asthma and atopic disorders.

**Methods:**

We searched the PubMed, Medline, Google Scholar, and EMBASE databases for RCTs published within the past 5 years (from 2017 to 2022). We included only full-text RCTs on human subjects published in or translated into the English language. We retrieved relevant data items with a preconceived data-extraction form and assessed the methodological quality of the selected RCTs using the Cochrane Collaboration's tool for assessing the risk of bias in randomized trials. We qualitatively synthesized the retrieved data to determine any significant differences in study endpoints of the probiotic and placebo groups.

**Results:**

A total of 1,320 participants (688 and 632 in the probiotic and placebo groups) from six RCTs were investigated. One RCT showed that early *Lactobacillus rhamnosus* GG (LGG) led to a reduction in the cumulative incidence rate of asthma. Another study demonstrated that mixed strains of *Lactobacillus paracasei* and *Lactobacillus fermentum* could support clinical improvement in children with asthma while one trial reported a significant reduction in the frequency of asthma exacerbations using a mixture of *Ligilactobacillus salivarius* and *Bifidobacterium breve*. Three trials showed that a combination of LGG and *Bifidobacterium animalis* subsp *lactis, Lactobacillus rhamnosus* alone, and a probiotic mixture of *Lactobacillus* ŁOCK strains improved clinical outcomes in children with atopic dermatitis and cow-milk protein allergy.

**Conclusions:**

Postnatal strain-specific probiotics (in single or mixed forms) are beneficial in preventing and treating atopic dermatitis and other allergies. Similarly, specific strains are more effective in preventing asthma or improving asthma outcomes. We recommend more interventional studies to establish the most useful probiotic strain in these allergic diseases.

## Introduction

Childhood asthma is a heterogeneous disease with several phenotypes and underlying endotypes. The heterogeneity is manifested in its various clinical features and the degree of airway inflammation and remodeling (1). As a result, precision medicine is considered essential for effectively managing the disease. Precision medicine refers to treatments tailored to meet individual patients' needs based on genetic, biomarker, phenotypic, or psychosocial characteristics that differentiate an index patient from other patients with similar clinical presentations (2). For instance, treatment of severe asthma has advanced from corticosteroids and bronchodilators to biologics like anti-immunoglobulin E (anti-IgE) medications (e.g., omalizumab) for patients with allergic background and anti-interleukin 5 (anti- IL5) medications (e.g., reslizumab) for those with eosinophilic background (3). Thus, precision medicine links phenotypes and endotypes to targeted therapies for better disease outcomes.

Recently, scientific searchlight has focused on the causal relationship between the human microbiome and these diseases (4–6). The human microbiome broadly refers to the aggregate of all resident microbiota, their anatomical sites of residence, and their collective genomes (7). The microbiota, which comprises commensal, symbiotic, and pathogenic microorganisms, are crucial for the human host's immunologic, hormonal, and metabolic homeostasis. Dysbiosis of gut and lung microbiota in infancy precedes the onset of asthma and atopic disorders later in childhood (8, 9).

Several randomized controlled trials (RCTs) now indicate that modifying lung and gut microbiota may serve as a preventive or treatment-optimization strategy in childhood asthma and atopic disorders such as atopic dermatitis, food allergy, and hay fever (10–13). This management approach is based on the bidirectional “cross-talk” between lung and gut microbiota. Given the importance of this gut-lung axis in sustaining immune balance (14), it is not surprising that intestinal and respiratory diseases show overlapping pathologic changes in the transition from gut inflammation to lung inflammation (15). For instance, patients with chronic inflammatory bowel diseases have a higher prevalence of inflammatory lung diseases (16). Disruptions in this bidirectional “cross-talk” across the gut-lung axis are associated with an increased risk of asthma in childhood (17).

Some environmental factors have a protective (positive) influence or an enhancing (negative) influence on the development of asthma (8). For instance, pollution, smoke, and pollen disrupt lung microbiota, whereas antibiotics and proton pump inhibitors (PPIs) interfere with gut microbiota. The resultant gut bacterial dysbiosis and reduced microbial diversity dysregulate the bidirectional “cross-talk” across the gut-lung axis and increase asthma prevalence (8, 14). On the other hand, exposure to the dairy-farming environment and probiotics are linked to lower incidences of asthma (8). Although reports about the efficacy of strain-specific probiotics in asthma prevention and treatment are conflicting, a meta-analysis of RCTs in six databases, however, revealed that the administration of *Lactobacillus rhamnosus* facilitated the prevention of asthma on subsequent follow-up (18). Additionally, several clinical trials suggest improved disease outcomes in asthma and allergic rhinitis (19, 20) and atopic dermatitis (21–28), when different strains of probiotics were either prenatally administered in pregnant women or postnatally administered in infancy and childhood.

Inflammation in asthma and atopic diseases is fundamentally mediated by T helper type 2 (Th2)-immune response (29). Production of several interleukins (IL) such as IL-4, IL-5, IL-9, IL-10, and IL-13 is specifically linked to Th2 cells. B lymphocytes respond to IL-4 stimulation by producing eosinophils and IgE antibodies which in turn enable mast-cell release of mediators of allergic responses namely histamine, serotonin, and leukotrienes. Gut microbiota plays a defined role in regulating immune function by, for instance, modulating Th1/Th2 balance as allergic diseases are associated with a tilt in this balance toward a Th2 response (30), or by directly stimulating Th17 differentiation (31). Since lung and gut dysbiosis occurs in asthma, probiotics potentially modify the bacterial dysbiosis, restore a physiologic immune response and reduce the associated Th2-mediated airway inflammation (6).

Previous systematic reviews and meta-analyses were conducted on RCTs of postnatal probiotics in the treatment of atopic dermatitis in children (32, 33), while others analyzed RCTs on prenatal and postnatal probiotics in the prevention of atopic dermatitis (34–37), and in the prevention or treatment of childhood asthma and wheeze (38, 39). Besides the focus on both prenatal and postnatal probiotics, most of the reviewed RCTs were published more than five years ago. Secondly, most of the systematic reviews have reported on the preventive or therapeutic outcomes in atopic dermatitis, with few analyzing only outcomes in asthma. Furthermore, the findings from these previous reviews appear inconsistent. More recent RCTs published within the past 5 years have linked specific probiotic strains to better disease outcomes and have focused more on postnatal probiotic supplementation than on giving probiotics to pregnant women. It is unclear if prenatal vs. postnatal probiotics are associated with different disease outcomes. Thus, the present systematic review was initiated as a new analysis of RCTs published within the last 5 years to determine if postnatal probiotics are as effective as prenatal probiotics in treating or preventing asthma and atopic disorders. We conducted and reported the review in adherence to the Preferred Reporting Items for Systematic reviews and Meta-analyses (PRISMA) guidelines (40).

## Methods

### Literature search strategy

We searched the PubMed, Medline, Google Scholar, and EMBASE databases. The search was focused on RCTs published within the last 5 years, i.e., from 2017 to 2022. (Date of final search: 29^th^ April 2022). Based on the title of the systematic review, the following descriptors were used in multiple combinations (as MeSH terms or not) with Boolean operators (AND/OR): “prevention and control”[Subheading] OR [“prevention”(All Fields) AND “control”(All Fields)] OR “prevention and control”[All Fields] OR [“prevention” (All Fields)] AND Optimizing[All Fields] AND [“therapy”(Subheading) OR “therapy”(All Fields) OR “treatment”(All Fields) OR “therapeutics”(MeSH Terms) OR “therapeutics”(All Fields)] AND “childhood”[All Fields] AND [“asthma”(MeSH Terms) OR “asthma”(All Fields)] AND Atopic[All Fields] AND [“disease”(MeSH Terms) OR “disease”(All Fields) OR “disorders”(All Fields)] AND Depend[All Fields] AND Alteration[All Fields] AND [“gastrointestinal microbiota” (MeSH terms)] OR [“gastrointestinal”(All Fields) AND “microbiota”(All Fields)] OR “gastrointestinal microbiota”[All Fields] OR [“gut”(All Fields) AND “microbiota”(All Fields)] OR [“gut microbiota”(All Fields)] AND [“probiotics”(MeSH Terms) OR “probiotics”(All Fields)].

### Eligibility and exclusion criteria

Eligible primary studies were full-text RCTs on human subjects published in or translated into the English language irrespective of each study's geographical location. Included studies were those published between 2017 and 2022. We excluded RCTs on experimental animal models, observational analytical studies (case-controlled, cohort, and cross-sectional studies), and other records published as abstracts, conference proceedings, reviews (narrative and systematic reviews/meta-analyses), editorials, letters to the Editor, and commentaries.

### Study selection

After screening the titles and abstracts of retrieved published articles, we independently evaluated potentially eligible full-text articles for final inclusion in the list of papers for the present systematic review. Duplicates and primary studies whose aims were not related to the aim of this systematic review were excluded during the selection process. We resolved possible disagreements on selected studies by reaching a consensus before selecting the eligible study.

### Quality assessment

We assessed the quality of the selected RCTs using the Cochrane Collaboration's tool for assessing the risk of bias in randomized trials (41). Risk-of-bias assessments were conducted on the following seven parameters: random sequence generation, allocation concealment, blinding of participants and personnel, blinding of outcome assessment, incomplete outcome data, selective reporting, and other bias. For each parameter, the risk-of-bias assessment was graded as low risk of bias- designated as (+), high risk of bias- designated as (-), and unclear risk of bias- designated as (?).

### Data extraction and data items

We used a preconceived data-extraction form to retrieve the following data items from the selected RCTs: author's name, year of publication, study setting, country of study, study population, including sample size and patients' demographics (age and sex), diagnosed childhood atopic disorder besides asthma, the intervention (type of probiotics administered) and the primary or secondary endpoints/outcome measures. Additionally, the risk of bias for each study was one of the extracted data items.

### Data synthesis

We assessed the study endpoints to establish if postnatal probiotics supplementation can reduce the incidence rates and improve the outcomes of childhood asthma and atopic disorders by modifying the gut microbiota. We qualitatively synthesized the retrieved data to determine any statistically or non-statistically significant differences in the outcome measures of the intervention (probiotics) groups and control (placebo) groups. For the qualitative and quantitative data, we evaluated categorical and numerical variables, respectively.

## Results

### Study selection

**Figure 1:**
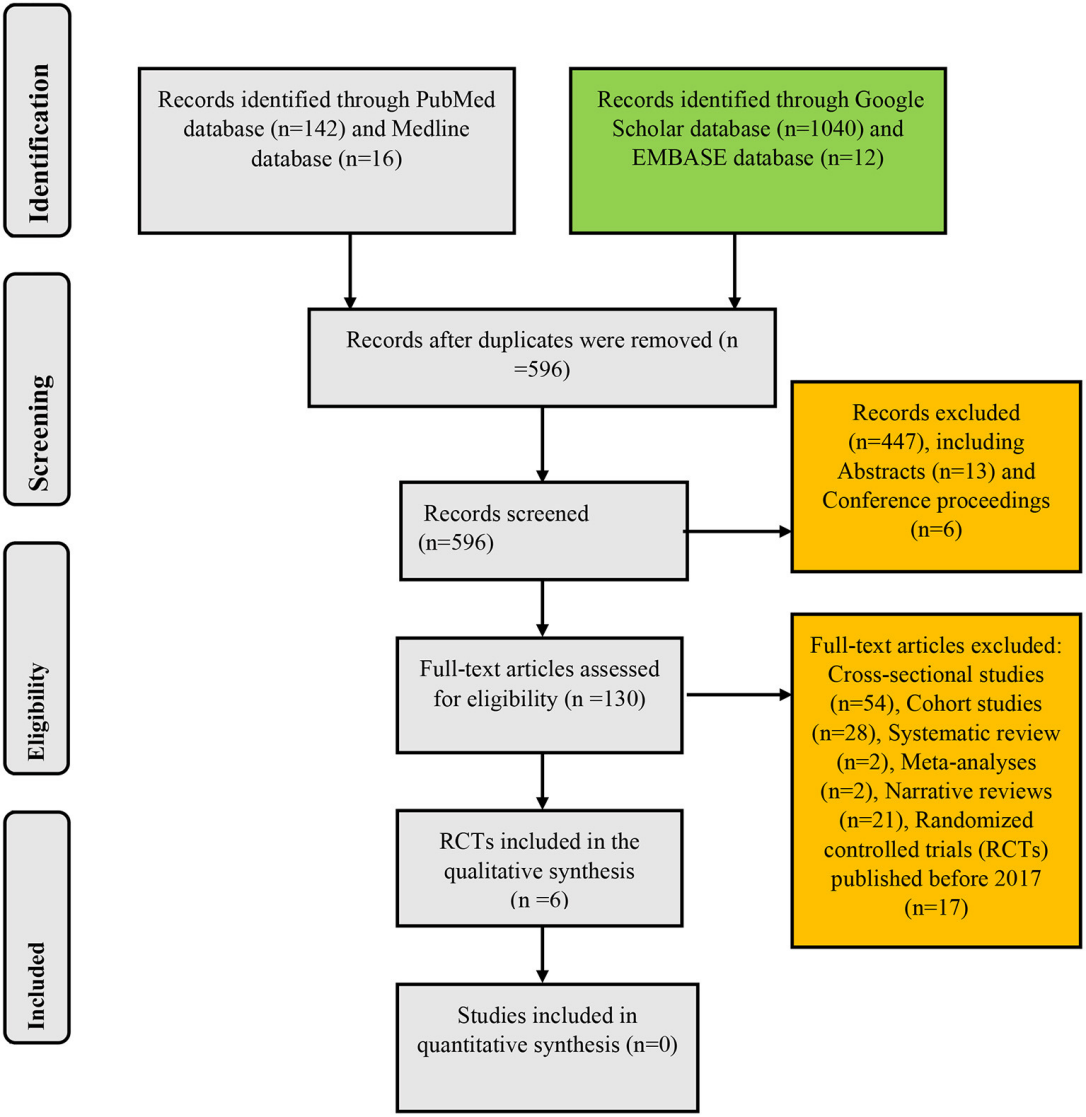
Algorithm for inclusion of randomized controlled trials on the use of probiotics in childhood asthma and atopic disorders.

The search of PubMed, Medline, Google Scholar, and EMBASE databases yielded 142, 16, 1,040, and 12 records, respectively: giving a total of 1,210 articles. After the removal of duplicates, the remaining records were 596. These remaining papers were then screened for their relevance to the present systematic review. This initial screening scaled down the number of papers to 149. Exclusion of cross-sectional studies (*n* = 54), cohort studies (*n* = 28), systematic reviews (*n* = 2), meta-analyses (*n* = 2), narrative reviews (*n* = 21), conference proceedings (*n* = 6), and abstracts (*n* = 13) yielded 23 RCTs. After limiting the study selection to articles published between 2017 and 2022, six papers that met the inclusion criteria were finally selected for analysis in the present systematic review. These selected RCTs were full-text articles published in the English language irrespective of the geographical setting of the studies (Figure 1).

### Study characteristics

As shown in Table 1, the six selected RCTs were all conducted in developed countries: one in the United States (11), two in Taiwan (19, 24), and three in the European countries of Denmark (12), Italy (42), and Poland (43). Two studies were conducted in community-based settings (11, 12), while four studies were conducted in hospital-based settings (19, 24, 42, 43). The total number of participants in the six RCTs was 1,320 (688 in the probiotics group and 632 in the placebo group). The participants' age distribution differed in five of the six trials with the following mean ages in the intervention (probiotics) and placebo groups, respectively: 9.98 ± 0.81 months and 10.08 ± 0.88 months (12), 7.68 ± 2.21 years/ 7.37 ± 2.34 years/ 7.00 ± 1.79 years and 7.86 ± 2.50 years (19), 1.5 ± 1.1 years and 1.8 ± 1.1 years for intent-to-treat (ITT) population and 1.4 ± 1.1 years and 1.8 ± 1.1 years for per-protocol (PP) population (24), 7.0 ± 3:38 years and 7.0 ± 2:95 years (42), and 8.2 ± 6.1 years and 8.8 ± 6.6 years (43). Their sex distribution showed an equal gender ratio in the study by Cabana et al. (11), and male predominance in the rest of the studies (12, 19, 24, 42, 43).

**Table 1:** Characteristics of the randomized controlled trials on probiotic administration for prevention or treatment-optimization of asthma and atopic diseases.

**Study**	**Country of study**	**Study setting**	**Study population (sample size and age/sex distribution)**
Cabana et al. (11)	United States	Community-based setting^*^ (San Francisco, California)	Intervention infants (*n* = 92) Control infants (*n* = 92) From infancy (4 days after birth) to 6 years Male/Female:44/48^†^ Male/Female:48/44^‡^
Schmidt et al. (12)	Denmark	Community-based setting**	Intervention infants (*n* = 143) Control infants (*n* = 142) 8–14 months^π^ Male/Female: 74/69^†^ Male/Female:71/71^‡^
Huang et al. (19)	Taiwan	Hospital-based setting (Pediatric outpatient clinics of the Taipei Hospital, Ministry of Health and Welfare Authority)	Intervention group (*n* = 112)^§^ Placebo group (*n* = 35) 6–18 years^ππ^ Male/Female:65/47^†^ Male/Female: 18/17^‡^
Wu et al. (24)	Taiwan	Hospital-based setting (Chung Shan Medical University Hospital and Taipei City Hospital)	Two parallel groups: ITT population [*N* = 66 (intervention group, *n* = 33 and placebo group, *n* = 33)] and PP population [*N* = 62 (intervention group, *n* = 30 and placebo, *n* = 32)] 4–48 months*** M/F:25/8 (ITT), 24/6 (PP)^†^ M/F:19/14 (ITT), 18/14 (PP)^‡^
Cukrowska et al. (43)	Poland	Hospital-based setting (multi-center study)	Probiotics group (*n* = 66) Placebo (*n* = 68) <2 years**** Male/Female:37/29^†^ Male/Female: 48/20^‡^
Drago et al. (42)	Italy	Pediatric primary-care setting	PP population [*N* = 422 (probiotics or active arm-*n* = 212 and placebo arm-*n* = 210)] Mean age 7.0 ± 3.17 years (Mean ages for active arm: 7.0 ± 3:38 years and placebo arm: 7.0 ± 2:95 years) Male/Female:121/91^†^ Male/Female:119/91^‡^

* Racially and ethnically diverse urban setting;^†^Sex distribution in the probiotic group;^‡^Sex distribution in the placebo group; **Danish-speaking community; ^π^Mean baseline ages in months: 9.98 ± 0.81 (intervention group) and 10.08 ± 0.88 (control group); ^§^Three intervention groups: Lactobacillus paracasei (LP) (n = 38), Lactobacillus fermentum (LF) (n = 38), and LP+LF (n = 36); ^ππ^Mean ages ± SD in years: 7.68 ± 2.21 (LP group), 7.37 ± 2.34 (LF group), 7.00 ± 1.79 (LP+LF group), and 7.86 ± 2.50 (placebo group); ITT, Intent-to-treat; PP, Per-protocol; ***Mean ages ± SD in years: 1.5 ± 1.1 (intervention group), 1.8 ± 1.1 (placebo group) for ITT population and 1.4 ± 1.1 (intervention group), 1.8 ± 1.1 (placebo group) for PP population; ****Mean ages ± SD in years: 8.2 ± 6.1 (probiotic group) and 8.8 ± 6.6 (placebo group).

**Figure 2:**
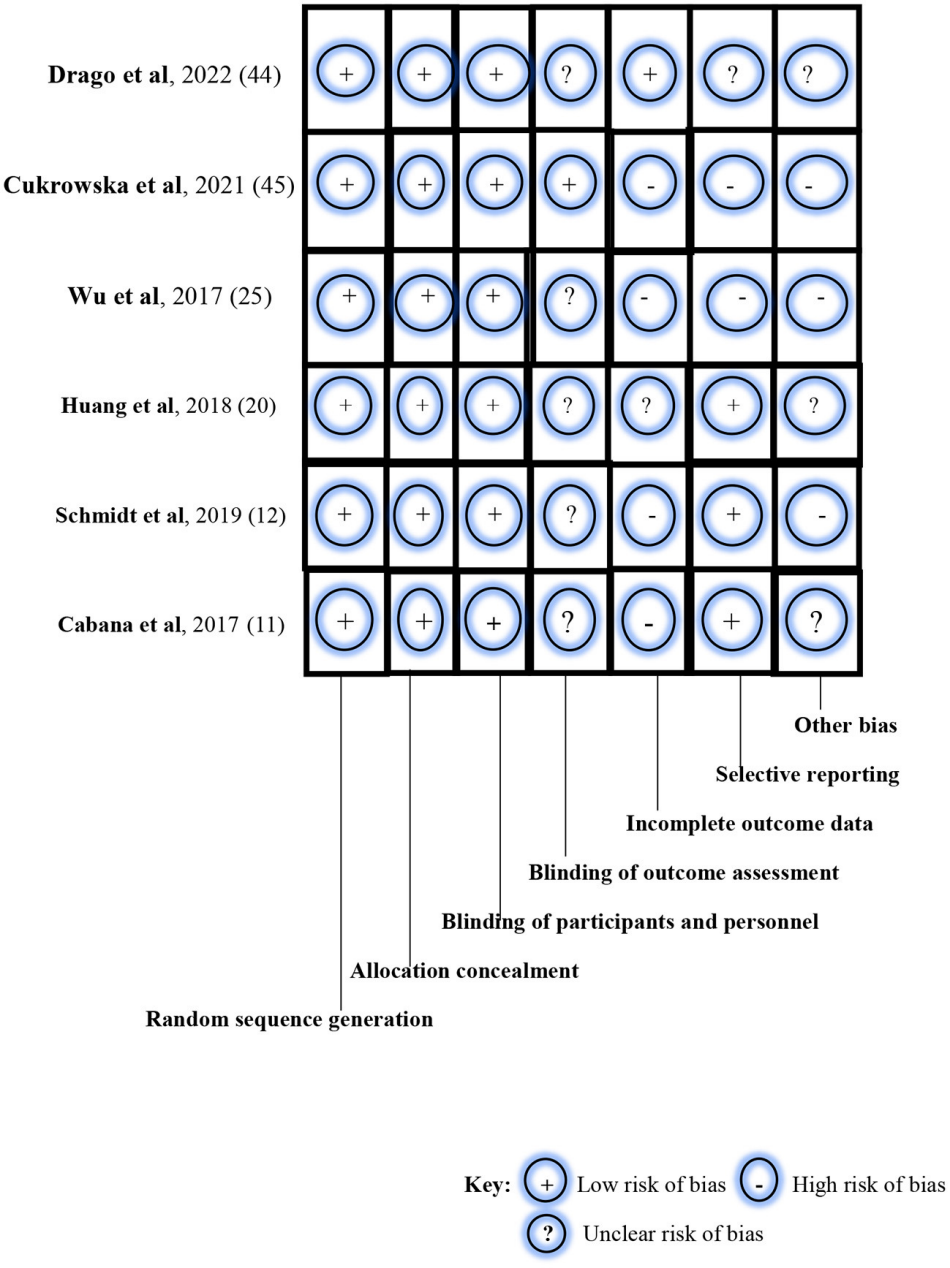
Risk-of-bias assessment of the randomized controlled trials using the Cochrane Collaboration's tool.

Using the Cochrane Collaboration's tool for assessing the risk of bias in RCTs, a low risk of bias was noted for random sequence generation, allocation concealment, and blinding of participants and investigators in all six studies (Figure 2). Specifically, block randomization was used in the study by Schmidt et al. (12) which ensured almost equal numbers of participants in each study arm: *n* = 143 (intervention group) and *n* = 142 (control group). The study by Cukrowska et al. (43) used a computer-generated randomization list which ensured that allocation sequences were easily concealed and not predictable. However, Wu et al. (24) in their randomization method simply reported that enrolled patients were either allocated to a treatment group or a control group at a ratio of 1:1; in the ITT population (*N* = 66), participants were thus equally distributed in the intervention group (*n* = 33) and the placebo group (*n* = 33). Similarly, Drago et al. (42) enrolled and randomized participants at the ratio of 1:1, using a computer-generated randomization method. Thus, their study participants were almost equally distributed in the probiotics arm (*n* = 212) and probiotics arm (*n* = 210). In the study by Cabana et al. (11), group allocation was also by a computer-generated randomization program whereas the study by Huang et al. (19) randomized the study participants using computer-generated 4-block design lists created by a statistician, with stratification based on demographics, disease severity, and current drug use.

In the blinding of outcome assessment, unclear risk of bias was noted in five studies (11, 12, 19, 24, 42), and low risk of bias in one study (43). However, a high risk of bias in incomplete outcome data was observed in four studies (11, 12, 24, 43), unclear risk of bias in one study (19), and low risk of bias in the remaining study (42). For example, the study by Schmidt et al. (12) reported the non-availability of outcome measurements among drop-outs after randomization and before baseline examination during the intervention. For selective reporting, a low risk of bias was noted in two studies (11, 19); high risk of bias was seen in three studies (12, 24, 43); and an unclear risk of bias was observed in one study (42). Specifically, selection bias was listed as one of the limitations in the study by Schmidt et al. (12). The study population was self-selected and consisted of participants with a special interest in the study. In the study by Wu et al. (24), enrolled participants were all children with atopic dermatitis who were grouped into either the ITT population or PP population. Similarly, the likelihood of selection bias was evident in the study of Cukrowska et al. (43) as targeted participants were children who had atopic dermatitis and cow-milk protein allergy. Also, parental consent and participation in the trial were driven by knowledge about the nature and outcome of the study. Finally, a high risk of bias was noted for other biases in three studies (12, 24, 43) and an unclear risk of bias in the remaining three studies (11, 19, 42). The studies by Schmidt et al. (12), Huang et al. (19), and Cukrowska et al. (43) particularly reported participant drop-outs during the trials, thus raising the likelihood of attrition bias.

### Study findings

Tables 2A,B summarize the major findings of the six RCTs. Firstly, the study by Cabana et al. (11) aimed to determine if a probiotic administration during the first 6 months of life reduces the incidence of childhood asthma and eczema in line with the trial of infant probiotic supplementation study (TIPS study). The TIPS study is a randomized, double-blind, parallel-arm, controlled trial designed to assess the effectiveness of daily *Lactobacillus rhamnosus* GG (LGG) supplementation for the first 6 months of life in decreasing the incidence of eczema (a potential early marker of asthma). The investigators administered daily capsules of 10 billion colony-forming units of LGG and 225 mg of inulin for the first 6 months of life to 92 infants in the intervention arm, and a 6-month course of daily capsules containing 325 mg of inulin to another 92 infants that served as controls. The primary outcome measure of their study was the incidence rate of eczema within 2 years of birth while the secondary outcome measures were the incidence rates of asthma and allergic rhinitis within 5 years of birth. Of the total of 184 participants observed over 6 years, eczema was diagnosed in 68 by 2 years of age. Asthma was diagnosed among 27 participants by the age of 5 years. Given the few participants with allergic rhinitis (*n* = 9), the authors did not analyze their cumulative incidence rate. Nevertheless, they found a cumulative incidence rate of eczema of 30.9% (95% CI, 21.4–40.4%) in the control arm and 28.7% (95% CI, 19.4–38.0%) in the LGG arm by the second year of life. Also, a cumulative incidence rate of asthma of 17.4% (95% CI, 7.6–27.1%) was noted in the control arm and 9.7% (95% CI, 2.7–16.6%) in the LGG arm by 5 years of age (Table 2A). Of the 27 study participants with asthma, 18 (67%) had an earlier diagnosis of eczema compared with 50 (32%) of 157 without asthma during the 6-year follow-up. Asthma risk was greater among participants with a previous history of eczema (Hazard Ratio 3.64; 95% CI, 1.66–7.96). Although the authors demonstrated a reduction in the cumulative incidence rate of asthma by 5 years of age, they concluded that early LGG supplementation for the first 6 months of life did not prevent the development of atopic dermatitis or asthma at 2 years of age for high-risk infants (11).

**Table 2A:** Major findings of the randomized controlled trials on probiotic administration for the prevention or treatment-optimization of asthma/atopic diseases.

**Study**	**Study aims/objectives**	**Study interventions**	**Study endpoints/outcomes**	**Major findings**
Cabana et al. (11)	To determine if a probiotic administration during the first 6 months of life reduces the incidence of childhood asthma and eczema (TIPS study)	A daily capsule of 10 billion colony-forming units of LGG and 225 mg of inulin for the first 6 months of life (for the intervention or probiotics arm) A 6 month course of daily capsules that contained 325 mg of inulin (for the control or placebo arm)	The incidence rate of eczema within 2 years of birth (primary outcome measure) Incidence rates of asthma and allergic rhinitis within 5 years of birth (secondary outcome measures)	The cumulative incidence rate of eczema of 30.9% (95% CI, 21.4–40.4%) in the control arm and 28.7% (95% CI, 19.4–38.0%) in the LGG arm by the second year of life The cumulative incidence rate of asthma of 17.4% (95% CI, 7.6–27.1%) in the control arm and 9.7% (95% CI, 2.7–16.6%) in the LGG arm by 5 years of age.
Schmidt et al. (12)	To determine the effect of LGG in combination with BB-12 administered in late infancy on the development of allergic diseases and sensitization (ProbiComp study)	Daily sachets of 1.0 g maltodextrin supplemented with 10 billion colony-forming units of LGG and 10 billion colony-forming units of BB-12 for 6 months (Intervention group) Daily sachets of 1.0 g maltodextrin only for 6 months (Placebo group)	The incidence rate of allergic diseases during the intervention period The incidence rate of sensitization using a specific IgE level of >0.35 PAU/L at the end of the intervention The incidence rate of food reaction during the intervention	At the mean follow-up age of 16.1 months (SD 0.9), eczema incidence rates of 4.2 and 11.5% in the probiotic group and the placebo group, respectively (*p* = 0.036) No difference in the incidence rates of asthma and conjunctivitis between the two groups
Huang et al. (19)	To determine the therapeutic effects of *Lactobacillus paracasei* (LP), *Lactobacillus fermentum* (LF), and their combination (LP + LF) on the clinical severity, immune biomarkers, and quality of life in children with asthma	Pure strains of *Lactobacillus paracasei* GMNL-133 (BCRC 910520, CCTCC M2011331) (LP), *Lactobacillus fermentum* GM-090 (BCRC 910259, CCTCC M204055) (LF), or their mixture (LP + LF) administered to three intervention groups and placebo to the placebo group, all for 3 months	Changes in GINA-based asthma severity and Childhood Asthma Control Test (C-ACT) scores over 3 months of the intervention compared with baseline (primary outcome measure). Changes in PAQLQ score, PASS, PEFR, skin prick test reactivity, serum immune biomarker levels, and fecal probiotic microbial composition (secondary outcome measures)	Compared with the placebo group, children receiving LP, LF, and LP + LF had lower asthma severity (*p* = 0.024, 0.038, and 0.007, respectively) but higher C-ACT scores (*p* = 0.005, <0.001, and <0.001, respectively). The LP + LF group demonstrated increased PEFR (*p* <0.01) and decreased IgE levels (*p* <0.05).

TIPS, Trial of infant probiotics supplementation; ProbiComp, Effect of Probiotics in Reducing Infections and Allergies in Young Children starting Daycare; LGG, Lactobacillus rhamnosus GG; BB-12, Bifidobacterium animalis subsp lactis; CI, confidence interval; GINA, Global Initiative for Asthma; PAQLQ, Pediatric Asthma Quality of Life Questionnaire; PASS, Pediatric Asthma Severity Scores; PEFR, peak expiratory flow rate.

**Table 2B:** Major findings of the randomized controlled trials on probiotic administration for the prevention or treatment-optimization of asthma/atopic diseases.

**Study**	**Study aims/objectives**	**Study interventions**	**Study endpoints/outcomes**	**Major findings**
Wu et al. (24)	To evaluate the efficacy and safety of *Lactobacillus rhamnosus* in children aged 4–48 months with atopic dermatitis	Allocation of enrolled patients into either a treatment (intervention) group [one capsule containing 350 mg *Lactobacillus rhamnosus* (MP108) and maltodextrin daily] or a control (placebo) group (one capsule of maltodextrin daily) at a ratio of 1:1, taken for 8 weeks	Comparison of the mean change of SCORAD after 8 weeks of treatment (primary efficacy endpoint) Comparison of the mean changes of SCORAD at post-baseline visits; the frequency and total amounts of the use of corticosteroids during the 8-week treatment; the frequency of AD and the symptom-free duration; the mean changes from baseline in IDQOL questionnaire at Week 4 and Week 8; and mean changes from baseline in DFI questionnaire at Week 4 and 8 (Secondary efficacy endpoints)	A significant difference in mean change in SCORAD from baseline of 21.69 ± 16.56 in the *Lactobacillus rhamnosus* group and 12.35 ± 12.82 in the placebo group for the ITT population* at week 8 (*p* = 0.014). A significant difference in mean change in SCORAD from baseline of 23.20 ± 15.24 in the *Lactobacillus rhamnosus* group and 12.35 ± 12.82 in the placebo group (*p* = 0.003) for the PP population** No difference in the dose of topical corticosteroids used in the two groups No significant difference in the overall symptom-free duration compared with the placebo group Significant improvement in IDQOL and DFI questionnaire scores at week 4 and 8
Cukrowska et al. (43)	To evaluate the effectiveness of the probiotic mixture of *Lactobacillus rhamnosus* ŁOCK 0900, *Lactobacillus rhamnosus* ŁOCK 0908, and *Lactobacillus casei* ŁOCK 0918 in children under 2 years of age with AD and a cow's milk protein (CMP) allergy	Administration of a mixture of three probiotic strains containing 1 billion (1 × 10^9^) colony-forming units (CFU) of these bacteria in the following proportions: 50% of *Lactobacillus casei* ŁOCK 0919, 25% of *Lactobacillus rhamnosus* ŁOCK 0908, 25% of *Lactobacillus rhamnosus* ŁOCK 090 and placebo (maltodextrin), all for 3 months with a subsequent 9 month follow-up	Changes in AD symptom severity assessed with the SCORAD index and Changes in the proportion of children with symptom improvement^‡^ (Primary outcomes) Level of total serum IgE and the presence of allergen-specific IgE (Secondary endpoint)	Significant decrease in SCORAD scores in both probiotic and placebo groups after 3 months (sustained after 9 months) The percentage of children showing symptom- improvement was significantly higher in the probiotic than in the placebo group after 3 months (OR = 2.56; 95% CI, 1.13-5.8; *p* = 0.012) Probiotic-induced improvement in SCORAD index mainly in allergen-sensitized patients (OR = 6.03; 95% CI, 1.85–19.67, *p* = 0.001)
Drago et al. (42)	To evaluate possible reduction of asthma flare-ups or exacerbations and improvement of disease severity using a mixture of *Ligilactobacillus salivarius* LS01 and *Bifidobacterium breve* B632 (the PROPAM study)	Administration of probiotic mixture of *Ligilactobacillus salivarius* LS01 (1 × 10^9^ live cells) and *Bifidobacterium breve* B632 (1 × 10^9^ live cells) or placebo (2 grams of maltodextrin) to enrolled patients twice daily for 8 weeks and subsequently once daily for a further 8 weeks	Reduction of asthma flare-ups, considering the number, duration (days), and severity of asthma attacks^†^ (primary outcome) Reduction of drugs used in maintenance and as-needed therapy for asthma flare-ups (secondary outcome)	Significant reduction in the number of asthma flare-ups by the probiotics mixture (OR = 3:17). The number of children with two asthma flare-ups was less than a third in the active (probiotics) group in comparison with the placebo group (OR = 3:65). For the severity of asthma flare-ups, children in the placebo group had 21 mild, 44 moderate, and 4 severe
				episodes while those in the active (probiotics) arm had 4 mild episodes, 19 moderate episodes, and 1 episode of severe asthma flare-up.

SCORAD, the score of atopic dermatitis; AD, atopic dermatitis; IDQOL, Infant Dermatitis Quality of Life; DFI, Dermatitis Family Impact; ITT, Intent-to-treat; PP, Per-protocol; *Patients who take at least one dose of study medication and have at least one efficacy measurement; ** A patient of ITT with a drug compliance rate over 80% PROPAM, probiotics in pediatric asthma management;^†^Severity was graded as mild, moderate and severe; CI, confidence interval; OR, odds ratio;^‡^A decrease in SCORAD score by at least 30% in comparison with that at baseline.

As part of the ProbiComp study (Effect of Probiotics in Reducing Infections and Allergies in Young Children starting Daycare), Schmidt et al. evaluated the effect of LGG in combination with *Bifidobacterium animalis* subsp *lactis* (BB-12) administered in late infancy on the development of allergic diseases and sensitization (12). They administered daily sachets of 1.0 g maltodextrin supplemented with 10 billion colony-forming units of LGG and 10 billion colony-forming units of BB-12 for 6 months to 143 participants aged 8–14 months. Daily sachets of 1.0 g maltodextrin were administered to 142 participants for 6 months who constituted the placebo group. The study endpoints were the incidence rate of allergic diseases during the intervention period, the incidence rate of sensitization using a specific IgE level of >0.35 PAU/L at the end of the intervention, and the incidence rate of food reaction during the intervention. These were determined with the following tools: doctor's diagnosis of allergic diseases, elevated specific IgE levels against a panel of food and inhalant allergens (in sensitized children), and parental observation and reportage of food reactions using web-based questionnaires. The major findings of the trial were the observation of eczema incidence rates of 4.2 and 11.5% in the probiotic group and the placebo group, respectively (*p* = 0.036) at mean follow-up age of 16.1 ± 0.9 months. However, there was no difference in the incidence rates of asthma and allergic conjunctivitis between the two groups (Table 2A).

Thirdly, the study by Huang et al. (19) aimed to determine the therapeutic effects of *Lactobacillus paracasei* (LP), *Lactobacillus fermentum* (LF), and their combination (LP + LF) on the clinical severity, immune biomarkers, and quality of life (QoL) in children with asthma. They administered unspecified doses of pure strains of *Lactobacillus paracasei* GMNL-133 (BCRC 910520, CCTCC M2011331) (LP), *Lactobacillus fermentum* GM-090 (BCRC 910259, CCTCC M204055) (LF), or their mixture (LP + LF) to three intervention groups and an unspecified placebo with unspecified dose to the placebo group, all for 3 months. The participants were distributed into the LP (*n* = 38), LF (*n* = 38), LP + LF (*n* = 36) and placebo groups (*n* = 35). Their ages ranged from 6 to 18 years. The investigators used changes in Global Initiative for Asthma (GINA)-based asthma severity and Childhood Asthma Control Test (C-ACT) scores over 3 months of the intervention compared with baseline as the primary outcome measure. Changes in Pediatric Asthma Quality of Life Questionnaire (PAQLQ) score, Pediatric Asthma Severity Score (PASS), peak expiratory flow rate (PEFR), skin prick test reactivity, serum immune biomarker levels, and fecal probiotic microbial composition were the secondary outcome measures. The authors found that children receiving LP, LF, and LP + LF had lower asthma severity (*p* = 0.024, 0.038, and 0.007, respectively) but higher C-ACT scores (*p* = 0.005, < 0.001, and < 0.001, respectively), compared with the placebo group. Additionally, the LP + LF group demonstrated increased PEFR (*p* < 0.01) and decreased IgE levels (*p* < 0.05) (Table 2A). Thus, LP, LF, or their combination (LP + LF) can support clinical improvement in children with asthma.

Furthermore, Wu et al. (24) evaluated the efficacy and safety of *Lactobacillus rhamnosus* in children aged 4–48 months diagnosed with atopic dermatitis. The authors allocated patients into either a treatment group who received one capsule containing 350 mg *Lactobacillus rhamnosus* (MP108) and maltodextrin, or a placebo group given one capsule of maltodextrin daily: all for 8 weeks (Table 2B). The patients were in two parallel groups: the ITT population [*N* = 66 (intervention group, *n* = 33 and placebo group, *n* = 33)] and the PP population [N=62 (intervention group, *n* = 30 and placebo, *n* = 32)]. The primary efficacy endpoint was the compared mean change of Score of Atopic Dermatitis (SCORAD) after 8 weeks of treatment. The secondary efficacy endpoints consisted of the comparison of the mean changes of SCORAD at post-baseline visits; the frequency and total amounts of the use of corticosteroids during the 8-week treatment; the frequency of atopic dermatitis and the symptom-free duration; the mean changes from baseline in Infant Dermatitis Quality of Life (IDQOL) questionnaire at Week 4 and 8; and mean changes from baseline in Dermatitis Family Impact (DFI) questionnaire at Week 4 and 8. The major findings of the trial consist of the following: a significant difference in mean change in SCORAD from baseline of 21.69 ± 16.56 in the *Lactobacillus rhamnosus* group and 12.35 ± 12.82 in the placebo group (ITT population) at week 8 (*p* = 0.014); a significant difference in mean change in SCORAD from baseline of 23.20 ± 15.24 in the *Lactobacillus rhamnosus* group and 12.35 ± 12.82 in the placebo group (PP population) at week 8 (*p* = 0.003); absence of differences in the dose of topical corticosteroids used in the two groups; absence of significant differences in the overall symptom-free duration compared with the placebo group; and significant improvements in IDQOL and DFI questionnaire scores at week 4 and 8. These findings strongly suggest that *Lactobacillus rhamnosus* was effective in ameliorating the symptoms of atopic dermatitis after an 8-week administration.

In the study by Cukrowska et al. (43), the researchers aimed to assess the effectiveness of the probiotic mixture of *Lactobacillus rhamnosus* ŁOCK 0900, *Lactobacillus rhamnosus* ŁOCK 0908, and *Lactobacillus casei* ŁOCK 0918 in participants aged <2 years diagnosed with atopic dermatitis and cow's milk protein allergy. They administered a mixture of three probiotic strains containing 1 billion (1 × 10^9^) colony-forming units (CFU) of these bacteria in the following proportions: 50% of *Lactobacillus casei* ŁOCK 0919, 25% of *Lactobacillus rhamnosus* ŁOCK 0908 and 25% of *Lactobacillus rhamnosus* ŁOCK 090 to the participants in the probiotics group (*n* = 66), and maltodextrin to those in the placebo group (*n* = 68); these interventions lasted for 3 months with subsequent 9-month follow-up (Table 2B). The primary outcomes were changes in symptom severity of atopic dermatitis assessed with the SCORAD index and changes in the proportion of children with symptom improvement. The level of total serum IgE and the presence of allergen-specific IgE were taken as the secondary endpoint. The results of the trial include a significant decrease in SCORAD in both probiotic and placebo groups after 3 months (sustained after 9 months); a significantly higher percentage of children showing symptom-improvement in the probiotic than in the placebo group after 3 months (OR = 2.56; 95% CI, 1.13–5.8; *p* = 0.012); and probiotic-induced improvement in SCORAD index especially in allergen-sensitized patients (OR = 6.03; 95% CI, 1.85–19.67, *p* = 0.001)- which was however not observed after 9 months. The study findings underscore the therapeutic benefits of the mixture of probiotic ŁOCK strains for children with atopic dermatitis and cow-milk protein allergy.

Finally, Drago et al. (42) evaluated the possible reduction of asthma flare-ups and improvement of disease severity using a mixture of *Ligilactobacillus salivarius* LS01 and *Bifidobacterium breve* B632 [the Probiotics in Pediatric Asthma Management (PROPAM) study]. They administered the probiotic mixture of *Ligilactobacillus salivarius* LS01 (1 × 10^9^ live cells) and *Bifidobacterium breve* B632 (1 × 10^9^ live cells) or placebo (2 g of maltodextrin) to enrolled patients (probiotics arm, *n* = 212, and placebo arm, *n* = 210) twice daily for 8 weeks and subsequently once daily for a further 8 weeks. They used reduction of asthma flare-ups (considering the number, duration in days, and severity of asthma attacks) as the primary outcome while the secondary outcome was the reduction of drugs used in maintenance and as-needed therapy for asthma flare-ups. The authors found that there was a significant reduction in the number of asthma flare-ups with the probiotics mixture (OR = 3:17). Also, the number of children with two asthma flare-ups was less than a third in the active (probiotics) group in comparison with the placebo group (OR = 3:65). For the severity of asthma flare-ups, children in the placebo group had 21 mild episodes, 44 moderate episodes, and 4 severe episodes while those in the active (probiotics) arm had 4 mild episodes, 19 moderate episodes, and 1 episode of severe asthma flare-up (Table 2B). This study has shown that these probiotic strains - *Ligilactobacillus salivarius* LS01 (DSM 22775) and *Bifidobacterium breve* B632 (DSM 24706) - were safe and significantly decreased the frequency of asthma flare-ups by more than a third.

## Discussion

The beneficial effects of prenatal or postnatal administration of probiotics in childhood asthma and atopic disorders, such as atopic dermatitis, have been well documented in several RCTs (44–48), although other RCTs reported contrary findings (49–52). Furthermore, evidence from previously published systematic reviews and meta-analyses strongly supports positive outcomes from either postnatal probiotics in the treatment of asthma, allergic rhinitis, and wheeze (18) or prenatal and postnatal probiotics in the prevention of atopic dermatitis (32–37). However, findings from other similar reviews show no unanimity regarding the same outcomes in the prevention of asthma and wheeze (38) or the prevention and treatment of asthma (39). In more recent RCTs which have linked better outcomes to specific probiotic strains, there appears to be a shift in the research focus to postnatal probiotics. A repeat systematic review of recent RCTs was, therefore, deemed necessary to answer the clinical question about whether postnatal probiotics are as effective as prenatal probiotics in improving outcomes in asthma and atopic disorders.

In the present systematic review, we analyzed and synthesized data from RCTs published within the past 5 years to determine the consistency of the findings reporting the positive therapeutic or preventive outcomes of probiotics in asthma and atopic disorders. We identified six RCTs that showed mixed reports on the benefits of probiotics in these diseases (11, 12, 19, 24, 42, 43). Generally, our review has demonstrated that different strains of bacterial probiotics (with postnatal administration of a single or mixed form) either reduced the incidence of or consistently improved the clinical outcomes in atopic dermatitis, cow-milk protein allergy, and asthma in most studies. Few studies, however, reported no influence on asthma outcomes. For instance, Cabana et al. reported that early probiotic supplementation with a *Lactobacillus* strain (LGG) for the first 6 months of life reduced the cumulative incidence rate of asthma by the fifth year of life although it failed to prevent the development of atopic dermatitis or asthma at the second year of life: particularly for high-risk infants (11). Similarly, Schmidt et al. (12) documented a significant reduction in the incidence rate of atopic dermatitis but not in that of asthma or allergic conjunctivitis when combined strains of probiotics (LGG and *Bifidobacterium animalis* subsp *lactis*) were administered early in infancy. On the other hand, Huang et al. demonstrated that clinical outcomes in asthma improved remarkably when affected children received the bacterial probiotic strains of *Lactobacillus paracasei* or *Lactobacillus fermentum* and their combination (19). While the study by Wu et al. showed that *Lactobacillus rhamnosus* attenuated the symptoms of atopic dermatitis (24), Cukrowska et al. (43) reported that combined probiotic ŁOCK strains (*Lactobacillus rhamnosus* ŁOCK 0900, *Lactobacillus rhamnosus* ŁOCK 0908, and *Lactobacillus casei* ŁOCK 0918) improved the clinical parameters in children with atopic dermatitis and cow-milk protein allergy. According to Drago et al. (42), other probiotic strains like *Ligilactobacillus salivarius* LS01 and *Bifidobacterium breve* B632 were especially effective in reducing the frequency of asthma exacerbations.

In a previous meta-analysis of seventeen RCTs by Du et al. (18), the pooled data for asthma risk after prenatal and postnatal probiotic supplementation showed no significant reduction compared with placebo groups whereas a subgroup of strains indicated that only *Lactobacillus rhamnosus* GG supplementation was significantly associated with a reduction in incident asthma. Another systematic review and meta-analysis of thirteen RCTs by Huang et al. (32) failed to robustly demonstrate the therapeutic benefits of probiotics in children with atopic dermatitis. Although SCORAD values were not significantly affected in studies conducted in Europe, they were significantly affected in Asian studies; again, *Lactobacillus rhamnosus* GG and *Lactobacillus plantarum* did not significantly affect SCORAD values in children with atopic dermatitis whereas *Lactobacillus fermentum* and *Lactobacillus salivarius* showed significant effects on SCORAD in these children (32). On the other hand, a meta-analysis of twenty-five RCTs by Kim et al. (33) found that therapy with a mixture of different bacterial species or *Lactobacillus* species showed greater benefit in children with atopic dermatitis than therapy with only *Bifidobacterium* species: a benefit not seen in infants with atopic dermatitis. Similarly, a meta-analysis of nine RCTs by Sun et al. (34) reported that the mixed strain of *Lactobacillus* and *Bifidobacterium* can effectively prevent incident atopic dermatitis in children under 3 years old. Whereas, a meta-analysis of sixteen RCTs by Mansfield et al. (35) demonstrated that prenatal and postnatal probiotics significantly reduced the incidence of atopic dermatitis in childhood, another meta-analysis of twenty-one RCTs by Tan-Lim et al. (36) showed that three probiotic mixtures were particularly effective in reducing the risk of atopic dermatitis; these probiotic combinations were Mix 8 (*Lactobacillus paracasei* ST11, *Bifidobacterium longum* BL999), LP (*Lactobacillus paracasei* subsp *paracasei* F19) and Mix 3 (*Lactobacillus rhamnosus* GG, *Bifidobacterium animalis* subsp lactis Bb-12). Furthermore, Zuccotti et al. (37) in their meta-analysis of seventeen RCTs reported that mixed probiotics significantly reduced atopic dermatitis risk in infants but failed to prevent the risks of asthma, wheezing, and rhinoconjunctivitis. The findings of these systematic reviews and meta-analyses are consistent with our findings which show that the effectiveness of probiotic supplementation in either atopic dermatitis or asthma is dependent on specific bacterial strains or their combinations. Based on this observation, we suggest that the mechanistic relationship between the gut and lung microbiota (gut-lung axis) on one hand and the gut and skin microbiota (gut-skin axis) on the other hand may explain the beneficial roles of probiotics in asthma, allergic rhinitis, and atopic dermatitis. The role of microbiota in regulating immune function has long been confirmed in studies that reported the modulation of Th 1/Th 2 balance by *Bacteroides fragilis* (30) and the induction of regulatory T cells (Treg) by *Clostridium* spp (53). Thus, children with atopic dermatitis are known to exhibit low biodiversity of their gut microbiota, especially the absence of *Bacteroides* diversity, and a high prevalence of gut colonization by *Clostridium difficile* (54, 55). Similarly, children with asthma show lung bacterial microbiota that is predominantly composed of *Clostridium* spp and *Bacteroides* spp (6). The microbiota plays a crucial role in the development of innate and adaptive immune responses which partly forms the basis for the “hygiene hypothesis” (56). This hypothesis suggests that lack of infectious exposure at a critical point in immune system maturation results in a greater risk for subsequent development of atopic disease and asthma (57). Bacterial dysbiosis, due to imbalances in the microbiota, contributes to delayed maturation of the immune system in children through the promotion of low levels of Th 1 cytokine response (58): leading to atopic diseases such as asthma, atopic dermatitis, allergic rhinitis, and food sensitization later in life (59, 60). These diseases are directly related to the balance between Th1- and Th2-linked cytokines as atopy-associated inflammation are mediated by Th 2 cytokine response. For instance, bacterial probiotics such as *Lactobacilli* and *Bifidobacteria* are administered to raise the levels of Th1 and reverse the Th2 imbalance (61): underscoring the fact that the immune-modulating effects of probiotics are strain-specific (62). These immune-modulating effects are mediated through bacterial-related components and bacteria-derived metabolites. The surface-associated exopolysaccharide of *Bifidobacterium longum* plays an important role in reducing host proinflammatory responses and inhibiting local Th 17 responses within the gut and the lung (63, 64), whereas short-chain fatty acids (SCFAs) produced by the gut microbiota or the probiotic bacterial strain-LGG-influence T cell responses by binding to G protein-coupled receptors (GPRs) in the lung (9). The ultimate sequel is the reduction in the incidence of asthma and other allergic diseases.

Contrary to the findings of our review that showed improved asthma outcomes in three studies (11, 19, 42), the systematic review by Azad et al. (38) found no robust evidence to show that perinatal probiotic supplementation was protective against doctor-diagnosed asthma or childhood wheeze. The authors, however, revealed that the twenty eligible RCTs they reviewed were heterogeneous in the type and duration of the probiotic supplementation, with most trials adjudged to be of high risk or unclear risk of bias due to attrition (38). Several probiotic organisms were evaluated singly or in combination, including four *Bifidobacterium* species (*B bifidum, B longum*, two strains of *B breve*, and four strains of *B lactis*), and six *Lactobacillus* species (*L acidophilus, L casei, L lactis, L reuteri*, two strains of *L paracasei* and three strains of *L rhamnosus*). Again, the systematic review of twelve RCTs by Jiang et al. (39) showed that probiotic supplementation was not significantly associated with a lower risk of asthma or wheeze: suggesting that it could not prevent these disorders in children. Nevertheless, six of the included studies indicated that probiotic supplementation improved lung function and asthma control in patients with asthma. Curiously, there are few similarities between the probiotic organisms used in the two studies we reviewed (*Lactobacillus paracasei* and *Bifidobacterium breve*) and those tested in some of the studies reviewed by Azad et al. (two strains of *Lactobacillus paracasei* and two strains of *Bifidobacterium breve*). We, therefore, suggest that apart from probiotic species, dose, and treatment duration, other determinants of disease outcomes may include the heterogeneity of the target population since there are differences in the composition of gut microbiota due to the geographical setting, method of delivery at birth and the type of infant feeding. For instance, babies delivered through the vaginal canal tend to have non-pathogenic, beneficial gut microbiota similar to those found in the mother (65), whereas the gut microbiota of babies delivered by Cesarean section comprises more pathogenic bacteria; developing non-pathogenic gut microbiota takes longer time in these babies (66). Thus, special recommendations had been made on the probiotic strains to be administered to infants born by Cesarean section. Some researchers demonstrated that *Limosilactobacillus reuteri* produced reuterin which removed gut pathogenic bacteria without harming other gut microbiota (67). Given the presence of *Limosilactobacillus reuteri* in human breast milk (68), the amount of this probiotic strain increases in maternal milk once orally administered to the mother, with the likelihood of being transferred to the baby (69). Thus, breast fed infants born by Cesarean section may have reduced microbial diversity with increased asthma prevalence (14). Concerning the type of infant feeding, the dysfunctional gut microbiota of breast fed infants (which also increases future asthma risk) can be normalized with early postnatal administration of the probiotic, *Bifidobacterium longum* subsp. *infantis*: with gut colonization persisting till the first year of life (70). In contrast, formula-fed infants tend to have reduced incidence of asthma (8).

Additionally, the present systematic review shows that all the RCTs were conducted in developed settings of the United States (11), Europe (12, 42, 43), and Taiwan (19, 24). We could not retrieve any eligible study conducted in the developing settings of sub-Saharan Africa in all the databases we searched. It could be due to either resource constraints (lack of funding) or dearth of expertise in conducting such trials, or both. Besides the relationship between industrialization-driven environmental pollution and prevalent atopy in childhood, the fact that geographical location may influence the composition of lung and gut microbiota warrants replication of similar studies in the developing world to correct the obvious inequities in research findings. The differences in cultural and nutritional habits in the developed and developing world may strongly contribute to disparities in the biodiversity of gut and lung microbiota of children in these settings. This observation further buttresses the importance of conducting RCTs on the effects of probiotics on childhood asthma and allergic diseases in sub-Saharan settings as well. For probiotics to be beneficial in asthma or allergic diseases, there is a consensus that the appropriate strain must be administered at the appropriate dose, at the appropriate timing or duration, and to the appropriate population (71, 72). For example, there are safety concerns about administering probiotics to preterm infants as they are at higher risk for adverse events such as bacteremia and sepsis (73).

This systematic review has some limitations. The number of reviewed RCTs was few because our search net was limited to publications within the past 5 years (2017-2022), and to studies that reported only postnatal probiotic supplementation. The majority of RCTs published before this period focused more on prenatal probiotics in the prevention or treatment of atopic dermatitis albeit with inconsistent findings. Because more recent RCTs focused on the preventive and therapeutic benefits of postnatal probiotics, we systematically reviewed the current evidence to determine if postnatal probiotics are as effective as prenatal probiotics in improving outcomes in asthma and other atopic disorders. We also aimed to establish if better disease outcomes are dependent on specific probiotic strains. Despite the high risk and unclear risk of bias for selective reporting and incomplete outcome data in most studies (with the likelihood of attrition bias), our findings are still in tandem with those of most reviews which concluded that probiotic supplementation was more beneficial in the prevention of atopic dermatitis and allergic rhinitis than in asthma and wheeze (38, 74–76), and which also suggested that postnatal supplementation with LGG may be specifically beneficial in preventing asthma, allergic rhinitis, and wheeze (18). Furthermore, we did not perform a robust meta-analysis because there was substantial heterogeneity between the studies which could have biased the summary effect size. For instance, the selected six studies showed differences in the ages of the participants, the probiotic strains, and the outcome measures.

Given the disparities in the disease outcomes following probiotic supplementation, we suggest that future research directions should focus on determining the following gray areas: the influence of geographic settings, the type of patients most likely to benefit from probiotics, probiotic species with better preventive and therapeutic effects, the effect of dosing on disease outcomes as well as the effect of probiotic bacterial composition on the pathogenesis of asthma. More recent studies suggest that preventive and therapeutic benefits were seen more in children aged > 1 year with atopic dermatitis (77). Similarly, interventions with mixed-strain probiotics [such as mix 8 (*Lactobacillus paracasei* ST 11, *Bifidobacterium longum* BL999), LP (*Lactobacillus paracasei* spp *paracasei* F19) and mix 3 (*Lactobacillus rhamnosus* GG, *Bifidobacterium animalis* spp *lactis* Bb-12)] have been found to result in better preventive and therapeutic outcomes in atopic dermatitis (36). Lastly, there should be an emphasis on using a homogeneous study population given the geographically-propelled differences in the composition of gut microbiota and possible individual responses to probiotic supplementation.

## Conclusions

In the present systematic review, current evidence shows that postnatal strain-specific probiotics (in single or mixed forms) are as effective as prenatal probiotics in the prevention or treatment of children with atopic dermatitis. Although the findings of similar reviews suggest some preventive or therapeutic benefits in asthma, our review confirms that administering certain probiotic strains is known to be effective in asthma prevention or in improving asthma outcomes. Specifically, our review shows that *Lactobacillus* strain (LGG) reduced asthma incidence rates whereas *Lactobacillus paracasei, Lactobacillus fermentum, Ligilactobacillus salivarius*, and *Bifidobacterium breve* improved asthma outcomes. While the findings of this review underscore the fact that postnatal probiotics are effective for the prevention and treatment-optimization of asthma, atopic dermatitis, and other allergic conditions, they also highlight the need for more interventional research to establish the most useful probiotic strain in these allergic diseases.

## Data availability statement

The original contributions presented in the study are included in the article/supplementary material, further inquiries can be directed to the corresponding author.

## Author contributions

SU and AA searched the electronic databases, retrieved published articles, and independently evaluated them for eligibility. Both authors assessed the methodological quality of the selected articles and extracted relevant data items. SU, AA, and JE synthesized the retrieved data. SU wrote the first manuscript draft. AA, JE, CO, CN, and IE criticized the draft and contributed to the subsequent draft. All authors approved the final draft.

## Conflict of interest

The authors declare that the research was conducted in the absence of any commercial or financial relationships that could be construed as a potential conflict of interest.

## Publisher's note

All claims expressed in this article are solely those of the authors and do not necessarily represent those of their affiliated organizations, or those of the publisher, the editors and the reviewers. Any product that may be evaluated in this article, or claim that may be made by its manufacturer, is not guaranteed or endorsed by the publisher.
